# Study on the Process of Intermolecular Forces and Electrostatic Force Between Cations and Nano-SiO_2_ Based on Molecular Simulation

**DOI:** 10.3390/molecules31142457

**Published:** 2026-07-14

**Authors:** Houjun Tang, Qiang Wang, Zheng Zhu, Jianhua Zhao, Yuxiang Sun, Feixiang Che, Meijuan Yuan, Meng Bao, Gang Liu, Weidong Li, Lei Zhang

**Affiliations:** 1 Research Institute of Shaanxi Yanchang Petroleum (Group) Co., Ltd., Xi’an 710065, China; 2 No. 11 Oil Production Plant, PetroChina Changqing Oilfield Company, Qingyang 745000, China; 3 School of Earth Resources, China University of Geosciences (Wuhan), Wuhan 430074, China

**Keywords:** nano-SiO_2_, cations, electrostatic force, van der Waals force, hydrogen bond

## Abstract

Although low-permeability oil reservoirs boast abundant resources, oil recovery remains relatively low due to the limitations of current water flooding development technology in oilfields. To address the current challenges of low-permeability oil reservoirs, nano-SiO_2_ particle aqueous solutions, instead of conventional water injection, have been applied to these reservoirs, which can achieve promising results. Nevertheless, due to the simple surface structure of nano-SiO_2_ particles, the unsaturated hydroxyl groups on their surfaces tend to undergo electrostatic attraction with cations in formation water, leading to particle aggregation and flocculation, ultimately compromising their stability. Therefore, studying the interaction between nano-SiO_2_ particles and cations in saline solutions is of great significance for providing guidance on the application of nano-SiO_2_ particles in low-permeability oilfields. In light of this, this paper employs molecular dynamics simulations and quantum chemical methods to investigate the processes of interactions between nano-SiO_2_ particles and cations from a microscopic perspective. The results indicate that the interaction zone between monovalent cations and nanoparticles lies approximately 0.2 nm to 0.3 nm away from the particle surface. In comparison, the interaction zone between divalent cations and nanoparticles extends roughly from 0.3 nm to 0.4 nm from the particle surface. The range and depth of influence of divalent cations are more pronounced. No covalent or ionic bonds are formed between monovalent cations and nanoparticles. However, divalent cations can form ionic bonds with nanoparticles, thereby altering their structural configuration. Among these interactions, electrostatic forces represent the dominant interaction force responsible for changing the configuration of nano-SiO_2_ particles, whereas van der Waals forces and hydrogen bonding forces are merely weak interactions. Moreover, as the valence state of the cation increases from monovalent to divalent, the cation forms new ionic bonds with the nano-SiO_2_ particles, significantly modifying their structural configuration and further undermining their stability. The findings of this study can improve our understanding of the existing state of nano-SiO_2_ particles in formation water, which can help to improve the application effect of nano-SiO_2_ particles in low-permeability oil fields.

## 1. Introduction

Currently, low-permeability oil and gas resources comprise a significant portion of global oil and gas reserves [1]. However, the development and utilization of these low-permeability resources face enormous difficulties and challenges [2]. Low-permeability reservoirs are characterized by poor petrophysical properties, low permeability, poor particle sorting, and high cementation content [3], all of which result in low recovery rates [4]. Therefore, developing cost-effective enhanced oil recovery (EOR) technologies specifically tailored for low-permeability reservoirs has become a critical issue that urgently needs to be addressed in the current exploitation of low-permeability oilfields [5]. To effectively improve the recovery rate of low-permeability reservoirs, nanotechnology-based EOR techniques have gradually come into focus [6]. In recent years, research and application of nanoparticles for enhancing oil and gas recovery have made considerable progress. The research shows that when nanomaterials are used as oil displacement agents [7], they can effectively reduce oil–water interfacial tension, and they can also be displaced into micropores to improve sweep efficiency due to their small size [8]. At the same time, they can also be adsorbed on the rock surface to reduce the water phase flow resistance, preventing the expansion of the rock after hydration, reducing the displacement pressure, and improving the water phase flow speed [9]. Therefore, nanomaterials can be applied to oil reservoirs in a variety of production methods to improve oil displacement efficiency, highlighting the huge development potential of nanomaterials in oilfield development.

At present, researchers have developed a variety of nanomaterials and technologies to improve crude oil recovery. There have been many pilot tests and field experiments on the application of nanomaterials in low-permeability oilfields [10]. Among the nanomaterials commonly used in trials are SiO_2_, NiO, Fe_3_O_4_, and MoS_2_. The experimental results consistently demonstrate that nano-SiO_2_ particles exhibit superior performance compared to other nanoparticles when applied to low-permeability reservoirs, primarily due to their more vigorous surface activity [11], lower cost, wider availability [12], and better dispersion properties [13]. Moreover, nano-SiO_2_ particles possess non-toxic and non-polluting characteristics, which not only ensure the safety of construction personnel but also contribute to environmental protection to some extent [14]. Therefore, using nano-SiO_2_ particles for enhanced oil recovery represents a relatively advanced and highly effective method for improving recovery rates [15]. Nano-SiO_2_ particles currently show promising application prospects in oilfield exploitation.

However, given the simple surface structure and high surface activity of nano-SiO_2_ particles, their excessive surface activity implies high chemical reaction energy, making them prone to agglomeration under complex reservoir conditions such as low permeability, high temperature, and high salinity, thereby causing pore blockage. Consequently, to achieve effective displacement and sustainable production of oil from reservoirs, it is crucial to develop efficient, low-impact, and cost-effective nano-SiO_2_ particle-based oil displacement agents for different reservoir conditions. This poses significant importance for the development of water flooding in low-permeability oil fields [16].

In recent years, with the rapid advancement of computer technology and the continuous refinement of related theories, molecular simulation has become a widely used and effective tool for researchers to study microscopic phenomena [17]. At the same time, molecular simulation enables experiments that are difficult or even impossible to conduct through macroscopic methods, providing microscopic data on intermolecular interactions and allowing us to elucidate, from a molecular perspective, the underlying mechanisms behind changes in the physicochemical properties of materials [18]. Therefore, by employing molecular simulation, we can investigate the interaction processes between nano-SiO_2_ particles and substances such as cations and hydrated ions present in oil reservoirs, analyze the physicochemical properties of nano-SiO_2_ particles, and examine how metal ions influence their structural configurations. This study can lay the foundation for future applications of nano-SiO_2_ particles in oilfield development [19]. Molecular simulation techniques encompass a variety of molecular simulation methods, but the two most commonly used are molecular dynamics simulations and quantum chemistry.

Using molecular dynamics simulation methods to study the migration and diffusion of molecules at solid–liquid, liquid–liquid, and gas–liquid interfaces has been a key area of early research in molecular dynamics simulations. During the process of investigating the interactions between nano-SiO_2_ particles and cations, as well as water molecules, molecular dynamics simulations allow us to explore the aggregation and diffusion states among molecules from a microscopic perspective [20]. From a molecular standpoint, this approach further elucidates how the aggregation of cations and anions on the surface of nano-SiO_2_ particles affects their physicochemical properties [21], thereby providing a theoretical foundation for the macroscopic application of nano-SiO_2_ particles in oil fields [22]. When studying interfacial properties at liquid–liquid interfaces, a method for constructing symmetric models was proposed, which is of great significance for calculating such properties and provides an important theoretical basis for constructing a model of nano-SiO_2_ particle interactions with salt solutions [23]. The transport properties of nanoparticles and the impact of charged nanoparticles on migration and aggregation were investigated by using molecular dynamics simulations [24]. The relationship between the charge of nanoparticles and stability at the two-phase interface was analyzed, demonstrating that when the charge of nanoparticles in solution reaches a certain threshold, it can enhance their stability in solution. After modification of nano-SiO_2_ particles, the particles exhibited an improved ability to reduce interfacial tension and an increased affinity between oil and water [25]. As previous studies have shown, molecular dynamics simulation is a method that explains intermolecular interactions at the molecular level, enabling quantitative analysis of molecular migration mechanisms and interaction processes. Therefore, from the perspective of molecular motion, molecular dynamics simulations can be employed to dissect the interaction processes between nano-SiO_2_ particles and cations, perform kinetic analyses, and explore changes in the interfacial properties of nano-SiO_2_ particles [26].

Molecular dynamics simulations can analyze the influence of molecular migration and aggregation behavior on interface properties through changes in momentum between molecules [25,27,28], while quantum chemistry can analyze the influence of molecules on the configuration of matter through changes in electron distribution [29]. The properties of rock surfaces, the molecular structures of oil substances, and pyrolysis mechanisms are studied using quantum chemical approaches, thereby revealing the relationship between changes in oil molecule structures and rock surface characteristics. The pore structure characteristics of the molecular model of the oil parent material are calculated by using quantum chemistry, and the transport model of oil and gas components in the oil parent material is also constructed by using quantum chemistry [30]. The interaction energy between hydrate cages and porous silica surfaces can be determined by employing quantum chemical calculations and examining the forces exerted by hydrate cages on silica at various facets and sites, as well as the types of these interactions. This explains how the formation and decomposition of various complexes affect the stability of the media surface [31]. Although research on the application of quantum chemistry to nano-SiO_2_ particles remains relatively scarce, previous studies have shown that quantum chemistry can be used to perform calculations on electronic structures and, based on electron distribution patterns, account for electrostatic interactions between materials [32]. Therefore, quantum chemical calculations can be used to analyze, from an electronic perspective, both the type and magnitude of interaction energies between nano-SiO_2_ particles and cations and to examine these interactions in light of the bonding states among molecules [33].

This paper employs molecular dynamics simulations and quantum chemical methods to explore the interactions between cations and nano-sized SiO_2_ particles. It quantitatively elucidates the effects of different force fields on their structural configurations [34,35,36]. In this study, we first construct an interfacial model of amorphous nano-sized SiO_2_ particles in salt solutions by using molecular dynamics simulations and investigate the interactions between various types of salt solutions (containing Na^+^, K^+^, Ca^2+^, and Mg^2+^) and nano-sized SiO_2_ particles. Subsequently, we use quantum chemical simulations to extract a portion of the surface from the molecular dynamics model and build models of approximately 1 nm diameter nano-SiO_2_ clusters interacting with individual cations (Na^+^, K^+^, Ca^2+^, and Mg^2+^), thereby examining the interactions at an even smaller scale [37,38,39]. The results of the research can be used to explain how different cations alter the physicochemical properties of nano-sized SiO_2_ particles, providing evidence for a change in the existing state of nano-sized SiO_2_ particles in formation water.

## 2. Molecule Simulation Experiment

### 2.1. Construction of the Model of Nano-SiO_2_ Particles and Cations

The conformational search and construction of nano-SiO_2_ cluster molecules were performed by using the two software packages: ABcluster software 2.0 and Gaussian View 6.0. Subsequently, quantum chemistry software, including Gaussian16, Multiwfn 3.5, and VMD 1.9.4, was used to calculate the parameters of the model of nano-SiO_2_ particles and cations [40,41,42]. The difference between quantum chemistry and molecular dynamics simulation is that the influence of the electron correlation effect on the system needs to be considered in the construction and calculation of the model. Through the method of quantum chemistry, part of the nano-SiO_2_ particle clusters in the molecular dynamics model were intercepted, and single cations (Na^+^, K^+^, Ca^2+^, Mg^2+^) were added to analyze the influence of cations and water molecules on the structure and properties of nano-SiO_2_ particles during the process of movement [43,44,45].

**Figure 1:**
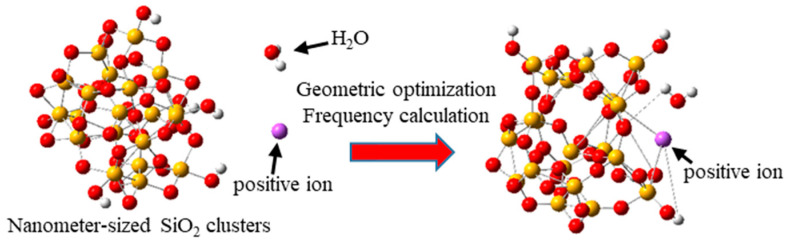
Molecular model of nano-SiO_2_ particles and a salt solution.

The construction and calculation of the nano-SiO_2_ particle–cation model involve the following four steps. First, by using Abcluster software, we search for cluster configurations of amorphous nano-SiO_2_ particles and visualize them in Gaussian View 6.0. Due to the limitations of the current technology of molecular simulation software, the modeling of nano-SiO_2_ clusters is achieved under relatively ideal conditions, which are smaller than actual nanoparticle clusters. However, the integrity of their structure is consistent with that of actual nano-SiO_2_ particles, and the constructed nano-SiO_2_ clusters are large enough compared to individual cations. Thus, this does not affect the trend of the simulation results. The radius of the nano-SiO_2_ clusters is approximately 0.5 nm, with a total of 69 atoms. These configurations include deprotonated siloxyl groups and hydroxylated siloxyl groups, reflecting the high surface activity and negative charge characteristics of amorphous nano-SiO_2_ particles under complex conditions [46]. Second, we perform geometric optimization of the nano-SiO_2_ cluster models by using Gaussian16 software to identify stable energy configurations. Third, we place individual ions and individual water molecules into the nano-SiO_2_ cluster models, with cations and water molecules positioned about 0.6 nm away from the surface of the nano-SiO_2_ clusters, thereby forming various types of nano-SiO_2_ particle–cation models, as shown in Figure 1. Finally, by using Gaussian16 software, we perform geometric optimization and frequency calculations for a total of 73 atoms across four different nano-SiO_2_ particle–cation models [47] (Na^+^, K^+^, Ca^2+^, and Mg^2+^), as illustrated in Figure 1.

When calculating the intermolecular forces in the nano-SiO_2_ particle–salt solution system, the analysis of the results will not take into account the influence of Cl^−^ ions for the following reason. Cl^−^ ions only diffuse at a distance far from nano-SiO_2_ particles, interacting weakly with nano-SiO_2_ particles. Existing studies have shown that when nano-SiO_2_ surfaces interact with solutions containing Na^+^, K^+^, Ca^2+^, and Mg^2+^, the silanol groups on the nano-SiO_2_ surface become negatively charged due to deprotonation, thereby exhibiting a more substantial adsorption effect on the cations in the solution. The nature of the involved cations primarily determines the magnitude of the interaction energy [48].

### 2.2. Parameters for Calculation

Based on the constructed nano-SiO_2_ particle–cation molecular model, quantum chemical calculations are performed, and the results are analyzed [49]. The parameters calculated are as follows. (1) The intermolecular forces between molecules are analyzed by using the simplified density gradient (RDG) function, and the strength and type of these intermolecular forces are interpreted. (2) The electrostatic force between molecules is analyzed by using the localized orbital localization (LOL) and independent gradient density (IGM) functions, which can interpret the strength of the electrostatic force and determine whether a bond is formed between the nano-SiO_2_ particles and the cations. The simulated temperature for the experiment was set at 300 K, and the pressure was set to normal pressure. The simulation time for molecular simulation was within the range of 0 to 1000 ps.

## 3. Results and Discussion

### 3.1. Analysis of Intermolecular Forces Between Nano-SiO_2_ Particles and Cations

During the process of the interaction between molecules, how do bonds form between them? And if bonds do form, how do the resulting intermolecular forces affect the structural stability of nano-SiO_2_ particles? To further elucidate the interaction forces between cations and nano-SiO_2_ particles, this section examines the weak interactions—specifically, intermolecular interactions between cations and the surface of nano-SiO_2_ particles—and explores the possibility of cations forming complexes with nano-SiO_2_ particles. In this section, the intermolecular interaction relationships among molecules are analyzed by utilizing Multiwfn software, and VMD software is used to generate spatial maps illustrating the regions of interaction between cations and nano-SiO_2_ particles [50].

**Figure 2:**
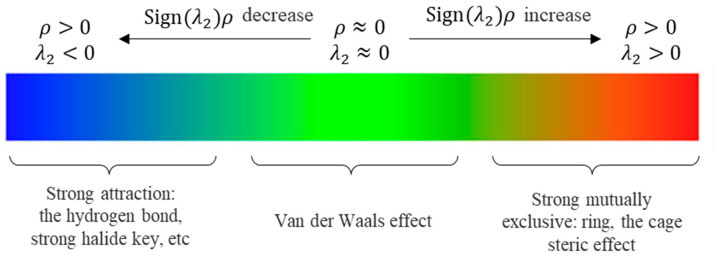
Relationship between ${Sign(\lambda }_{2})$ and ρ(r) functions and the types of molecular interactions.

In the study, an intermolecular interaction approach was employed. By using the simplified density gradient (RDG) function, RDG isosurface maps of regions with low electron density were generated to highlight areas associated with various noncovalent interactions visually [51]. The simplified density gradient (RDG) is a dimensionless form of the electron density gradient, serving as an indicator of the strength of weak interactions. Furthermore, the type of weak interaction is represented by the functional relationship between ρ(r) and ${Sign(\lambda }_{2})$. The corresponding interaction types are illustrated in Figure 2.

**Figure 3:**
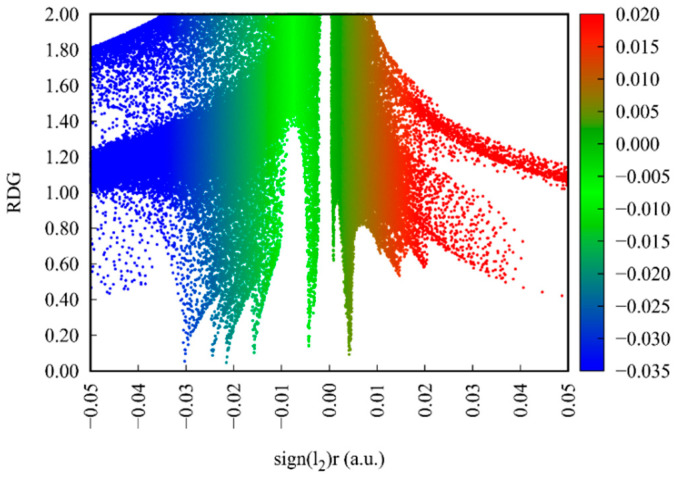
Color-filled scatter plot of Na^+^ ion RDG.

**Figure 4:**
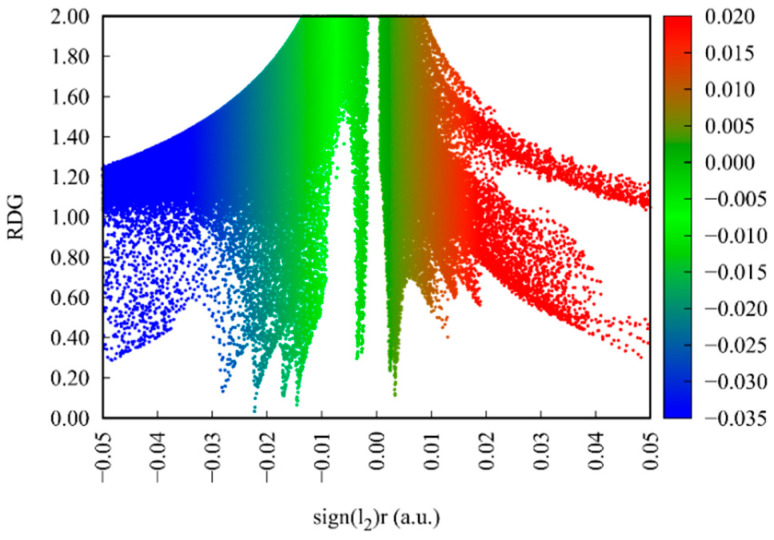
Color-filled scatter plot of K^+^ ion RDG.

**Figure 5:**
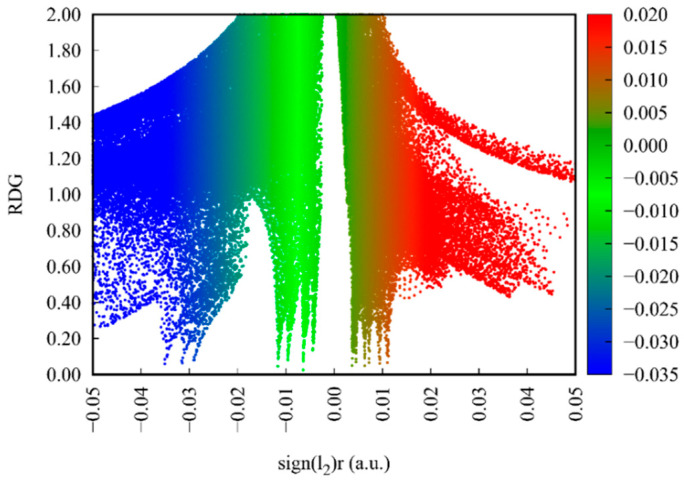
Color-filled scatter plot of Ca^2+^ ion RDG.

**Figure 6:**
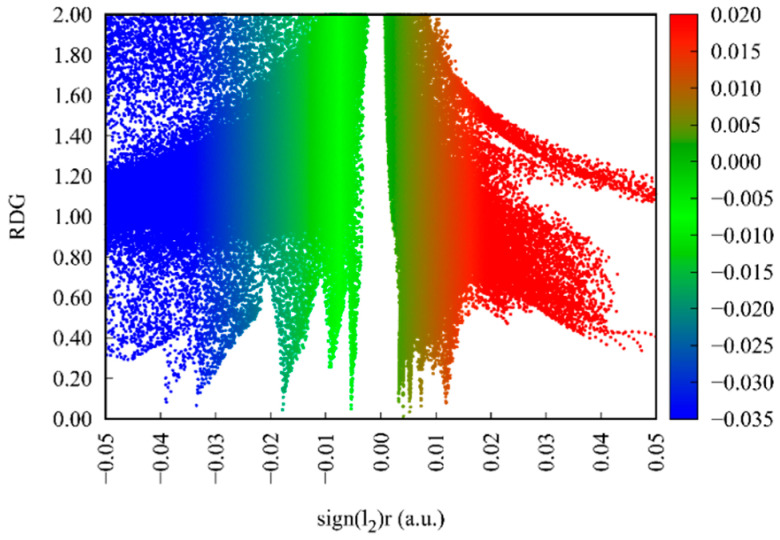
Color-filled scatter plot of Mg^2+^ ion RDG.

In order to highlight the intermolecular interactions among cations, nano-SiO_2_ particles and water molecules, the interactions within nano-SiO_2_ particles were ignored when using RDG diagrams. The computational results are shown in Figure 3, Figure 4, Figure 5 and Figure 6. The figures show RDG scatter plots colored according to the concentrations of Na^+^, K^+^, Ca^2+^, and Mg^2+^ ions. On the left, the y-values represent the magnitude of the RDG(r) function values. On the right, the y-values indicate the type and magnitude of molecular interactions, with colors corresponding to these values. The x-values represent the magnitude of the product of the ${Sign(\lambda }_{2})$ function and the ρ value.

Figure 3 and Figure 4 show RDG scatter plots colored by density for Na^+^ and K^+^. As can be seen from the horizontal axis of the RDG scatter plots, when x is in the range of (−0.02, 0), this region corresponds to the area of weak intermolecular interactions, primarily governed by van der Waals forces. In the Na^+^ RDG scatter plot, the range of van der Waals interactions is slightly larger than that for K^+^. When x is in the range of (−0.05, −0.02), this region corresponds to the vicinity of the atomic nuclei, where hydrogen bonding plays the dominant role. In the Na^+^ RDG scatter plot, the range of hydrogen bonding is greater than that for K^+^. When x is in the range of (0, 0.05), both Na^+^ and K^+^ exhibit relatively weak steric effects with respect to the nanoparticles. By examining the isosurfaces of molecular configurations, it can be observed that van der Waals interactions occur between monovalent cations, water molecules, and nanoparticles. Additionally, hydrogen bonding occurs between cations and water molecules, as well as between nanoparticles and water molecules. Among these interactions, the interaction between monovalent cations and water molecules is stronger than that between nanoparticles and water molecules. From the extent of these interactions, as shown by the isosurfaces of molecular configurations, it is evident that the interactions among monovalent cations, water molecules, and nanoparticles are confined to a tiny region—approximately 0.2 nm to 0.3 nm—indicating that the intermolecular interactions between monovalent cations and water have minimal influence on nanoparticles. Consequently, it is unlikely that monovalent cations can form stable complexes with nanoparticles.

Figure 5 and Figure 6 show RDG scatter plots colored by density for Ca^2+^ and Mg^2+^. As can be seen from the horizontal coordinates of the RDG scatter plots, when x = (−0.02, 0), the van der Waals interaction range in the Ca^2+^ RDG scatter plot is slightly smaller than that in the Mg^2+^ plot. When x = (−0.05, −0.02), the hydrogen bonding interaction range in the Ca^2+^ RDG scatter plot is significantly smaller than that in the Mg^2+^ plot. When x = (0, 0.05), there is no significant difference between Ca^2+^ and Mg^2+^ in terms of steric effects with the nanoparticles. However, both ions form a common equipotential surface with van der Waals interactions, indicating a relatively pronounced steric effect. By examining the molecular configuration of equipotential surfaces, it can be observed that both van der Waals interactions and steric effects exist between the divalent cations, water molecules, and nanoparticles, while hydrogen bonding occurs between the cations and water molecules as well as between the nanoparticles and water molecules. Notably, the van der Waals interaction range and the hydrogen bonding interaction range of Mg^2+^ are both larger than those of Ca^2+^. From the perspective of interaction ranges, Mg^2+^ exhibits a broader interaction range with the nano-SiO_2_ particles, to some extent reflecting a stronger interaction force. Analysis reveals that the primary types of intermolecular interactions between molecules are van der Waals forces and hydrogen bonds. Compared to monovalent cations, divalent cations exhibit a more substantial steric hindrance effect when interacting with nano-SiO_2_ particles, leading to more pronounced local energy changes in nano-SiO_2_ particles. Furthermore, judging from the number of scatter points, Mg^2+^ ions have a broader range of action than Ca^2+^ ions, yet both ranges are wider than those of monovalent cations, indicating that divalent cations are more likely to form covalent or ionic bonds with nano-SiO_2_ particles.

### 3.2. Analysis of Electrostatic Force Between Nano-SiO_2_ Particles and Cations

The electrostatic interaction among cations and nano-SiO_2_ particles is examined by using the Localized Orbital Locator (LOL) and the Independent Gradient Model (IGM). The bonding mechanism between cations and nano-SiO_2_ particles has been analyzed [52]. In the studies by Schmider and Becke [53,54], a functional relationship was established between the local kinetic energy density $t_{\sigma }$ and the finite variable $v_{\sigma }$, and the Localized Orbital Locator (LOL) was proposed to assess the strength of intermolecular interactions and determine whether bonds are formed [53]. In the study by Lefebvre et al. [55], a function was defined based on the gradient of regional atomic density, which calculates the density difference between two atoms and constructs an isosurface model. Finally, the degree of mutual interference between atoms was analyzed according to the magnitude of this density difference. Compared with the Localized Orbital Locator, the Independent Gradient Model has the advantage of better representing the interactions between atoms in two-dimensional systems. Based on this, the process of electron exchange kinetic energy between molecules can be identified by mapping the color variations of the $v_{\sigma }$ function in an electron localized orbital diagram and then determining whether covalent bonds have formed between molecules by plotting isosurfaces of the Independent Gradient Model.

**Figure 7:**
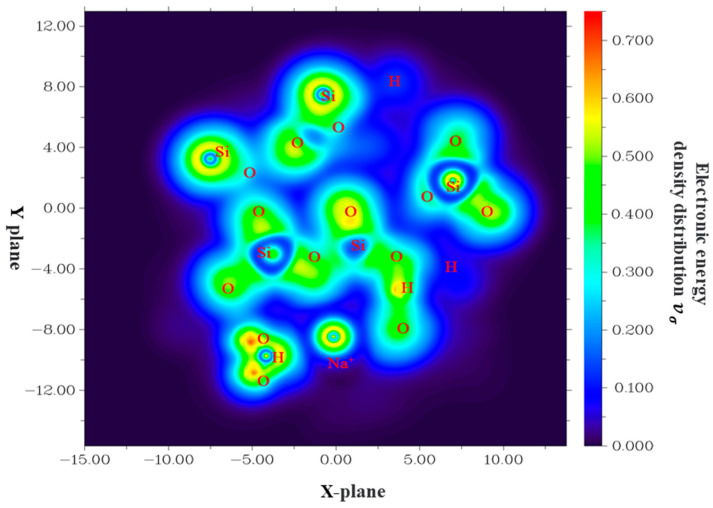
Regional orbital diagram of Na^+^ ions.

**Figure 8:**
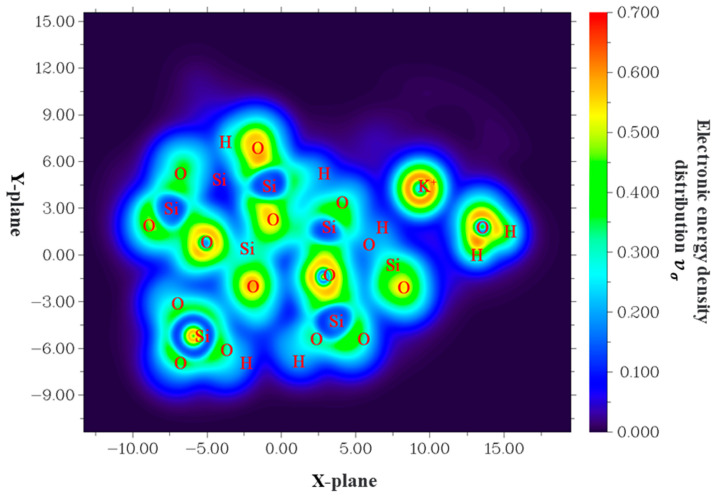
Localized orbital diagram of K^+^ ions.

**Figure 9:**
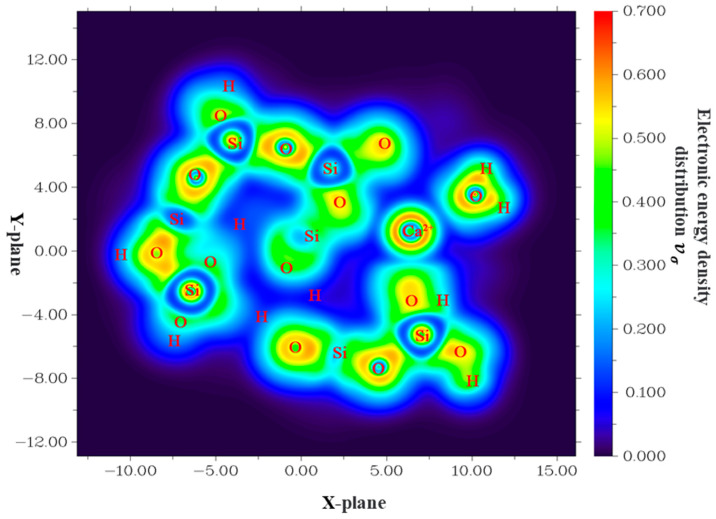
The regional orbitals of Ca^2+^ ions.

**Figure 10:**
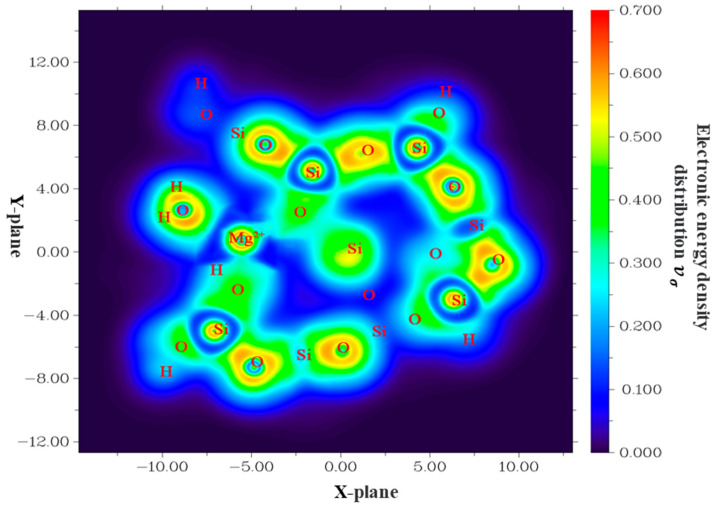
Regional orbital diagram of Mg^2+^ ions.

Figure 7, Figure 8, Figure 9 and Figure 10 show planar representations of molecular localized orbitals. It can be observed that the planar configurations of the nano-SiO_2_ particles are inconsistent. This inconsistency arises from the varying relative positions of the cations. Accordingly, the molecular plane is defined with the *Z*-axis in the range (−2, 2) and the XY-axis serving as the horizontal and vertical coordinates. Such a setup enables a more accurate representation of the covalent interactions between the cations and the nano-SiO_2_ particles. In the figures, the *x*-axis represents the coordinates of the X-plane, while the left *y*-axis indicates the coordinates of the Y-plane. Thus, the three-dimensional molecular system is plotted on the XY-plane to generate a planar representation of molecular localized orbitals. The right *y*-axis shows the values of the finite variable $v_{\sigma }$, with different values corresponding to other colors. The color sequence, from blue to green to red, corresponds to increasing values from small to large. When $v_{\sigma }$ = 0, it signifies that no electron exchange occurs in this region, implying no change in energy. When $v_{\sigma }$ = (0, 0.7), it indicates that as the value of c increases, the electron kinetic density rises, leading to more frequent electron exchanges among molecules and stronger covalent interactions.

First, by comparing the localized orbital plots of Na^+^ and K^+^ ions (Figure 7 and Figure 8), it can be observed that the electron density regions between Na^+^ and K^+^ ions and the nano-SiO_2_ particles range from 0 to 0.2, with colors transitioning from blue to cyan–green, indicating a weak electron exchange interaction in both cases. However, from the perspective of cation integrity, there are no apparent gaps in the nuclear regions, and the electrons are strongly localized, with slight variation in local electron energy density. This suggests that neither Na^+^ nor K^+^ ions form covalent or ionic bonds with nano-SiO_2_ particles. From the perspective of the electron density region between water molecules and nano-SiO_2_ particles, the $v_{\sigma }$ value between water molecules and nano-SiO_2_ particles in the Na^+^ ion localized orbit is 0.2~0.3, and the $v_{\sigma }$ value between water molecules and nano-SiO_2_ particles in the K^+^ ion localized orbit is 0~0.2. This indicates that water molecules in the Na^+^ and K^+^ ion localized orbits may form weak hydrogen bonds with nano-SiO_2_ particles, but the strength of the hydrogen bonds is different. From the perspective of the electron density region between Na^+^ ions, K^+^ ions, and water molecules, the value of $v_{\sigma }$ is 0~0.1, indicating that the electron exchange density between Na^+^ ions, K^+^ ions, and water molecules is weak. Therefore, apart from the possibility that water molecules may form weak hydrogen bonds with nano-SiO_2_ particles, thereby exerting some interaction energy on these particles, the local electron kinetic density changes between Na^+^ ions, K^+^ ions, nano-SiO_2_ particles, and water molecules are minimal. Moreover, the ring-shaped regions around the cations show no obvious gaps, and no covalent or ionic bonds have formed between Na^+^ ions, K^+^ ions, and nano-SiO_2_ particles.

Next, by comparing the localized orbital plots of Ca^2+^ and Mg^2+^ ions (Figure 9 and Figure 10), it can be found that the electron density regions between Ca^2+^ and Mg^2+^ ions and the nano-SiO_2_ particles have a $v_{\sigma }$ value ranging from 0.3 to 0.45, with colors transitioning between cyan and green, indicating significant electron exchange interactions in both cases. From the perspective of cation integrity, the ring-shaped regions associated with each cation exhibit small gaps, suggesting strong electron delocalization and substantial local variations in electron density. This could imply the formation of either covalent or ionic bonds between the cations and unsaturated siloxyl groups within the nano-SiO_2_ particles. Compared to Ca^2+^ ions, Mg^2+^ ions show a greater degree of overlap with the electron density regions of the nano-SiO_2_ particles, indicating that Mg^2+^ ions undergo more frequent electron exchanges with the nano-SiO_2_ particles and experience larger local energy fluctuations than Ca^2+^ ions. Looking at the electron density regions between water molecules and the nano-SiO_2_ particles, it can be observed that the $v_{\sigma }$ values for water molecules in the localized orbitals of both Ca^2+^ and Mg^2+^ ions range from 0 to 0.2, showing little difference between the two. This suggests that water molecules in the localized orbitals of both Ca^2+^ and Mg^2+^ ions can potentially form relatively weak hydrogen bonds with nano-SiO_2_ particles, with no significant difference in the strength of these hydrogen bonding interactions. Furthermore, examining the electron density regions between Ca^2+^ and Mg^2+^ ions and water molecules, it can be found that the $v_{\sigma }$ values range from 0 to 0.1, with a fairly distinct boundary, indicating weaker electron exchange densities between Ca^2+^ and Mg^2+^ ions and water molecules. Therefore, it is plausible that both Ca^2+^ and Mg^2+^ ions may form covalent or ionic bonds with the unsaturated siloxyl groups within nano-SiO_2_ particles, while water molecules also exert a certain degree of weak hydrogen bonding interaction with nano-SiO_2_ particles.

When comparing monovalent cations (Na^+^ and K^+^) with divalent cations (Ca^2+^ and Mg^2+^), the electron exchange interactions between monovalent cations and nano-SiO_2_ particles are infrequent, and no covalent or ionic bonds are formed. In contrast, the electron exchange interactions between divalent cations and nano-SiO_2_ particles are much more frequent, potentially leading to the formation of covalent or ionic bonds. This indicates that the covalent interaction between divalent cations and nano-SiO_2_ particles is stronger. As the number of divalent cations increases, there is a high likelihood that the molecular configuration of nano-SiO_2_ particles will be altered. From the degree of electron exchange between the four types of cations and the nano-SiO_2_ particles, it is evident that the covalent interaction between divalent cations (Ca^2+^ and Mg^2+^) and nano-SiO_2_ particles is stronger than that between monovalent cations (Na^+^ and K^+^).

## 4. Conclusions

The intermolecular forces between cations and nano-SiO_2_ particles mainly include van der Waals forces, hydrogen bonding, and steric effects. They primarily manifest as van der Waals forces and hydrogen bonding. As the valence of the cation increases, the intermolecular forces between the cation and nano-SiO_2_ particles are slightly enhanced. Specifically, divalent cations exhibit a wider interaction range and deeper influence than monovalent cations, forming a strong steric hindrance effect. Consequently, divalent cations are more likely to form complexes with nano-SiO_2_ particles. For electrostatic interactions, the electron exchange between monovalent cations and nano-SiO_2_ particles is infrequent, resulting in the absence of covalent or ionic bonds. However, ionic bonds are formed between divalent cations and nano-SiO_2_ particles, altering the configuration of nano-SiO_2_ particles to some extent. Based on the aforementioned study, the key to the destabilization of nano-SiO_2_ particles in salt solutions lies in electrostatic interactions. Identifying methods to reduce electrostatic interactions between nano-SiO_2_ particles and salt solutions is crucial for mitigating flocculation and agglomeration of nano-SiO_2_ particles and enhancing their efficient application in the field of oil development.

## Data Availability

The data will be made available upon request.
